# RSC in practical use

**DOI:** 10.1186/1748-7161-8-S2-P7

**Published:** 2013-09-18

**Authors:** Alem Terzic

**Affiliations:** 1 Schreiber und Ebert GmbH, Frankfurt, Germany

## 

The goal of this study was to determine the primary correction of the Cobb angles of idiopathic scoliosis subjects wearing the Rigo System Cheneau (RSC) brace. The RSC brace is a scoliosis brace that incorporates expansion and pressure areas to treat all aspects of the 3-dimensional scoliotic deformity not only in the frontal plane but also in the sagittal and transverse planes. 

After years of treatment without looking at the interdisciplinary way of bracing, we decided to change our method of bracing. There are different philosophies and design possibilities in the conservative treatment of idiopathic scoliosis (IS) and lots of CPO’s worldwide have an opinion about the best brace or best method to treat IS. 

It is important that the goals of treatment are clearly defined and every effort is made to prevent the progression of the scoliotic deformity throughout treatment. Some braces can not only be ineffective, but may be harmful. . In certain cases, damage occurs if the patient is wearing an ill-fitting brace or if the treatment is not combined with physiotherapy. Often, the patients and their families are not provided information or assistance with the subsequent diagnosis and therapy. Sometimes, patients and their families contact doctors who do not have much experience with treating IS. Often, patients do not recognize the scoliosis until it is more severe, so treating the scoliosis becomes a more serious problem. However, the importance of providing adequate scoliosis information and a solid treatment plan for scoliosis physical therapy has recently become recognized. In Germany, especially, which has its own unique reimbursement system, the CPO’s are not pressured to improve their quality or way of treatment. Often, these CPOs are paid by health insurance providers without adequate follow-up, or the braces are not appropriate or do not conform to SOSORT guidelines. 

Until now, there was no institution to prove or allow the bracing or any CPO treatment in Germany because of missing studies of properly fitted braces and treatment. It is one thing to provide a patient with a prosthetic leg with numerous issues with the socket, but it is an entirely different issue to provide an ill-fitting brace to an infant or adult patient without concept of treatment. 

Scoliosis is a multifactorial deformity that affects all three body planes of the trunk and spine. It presents a lateral curvature with torsion of the spine and chest, often associated with abnormal sagittal profile, such as flatback. I want to discuss the brace form or correct manner of modelling the brace, which is surely very important; however, the full concept, such as RSC, is also very important in providing good results. Since 2001, the RSC Management System is a patented method for producing computer-standardized custom-molded scoliosis orthoses for patients with scoliosis. This brace system is based off handmade molds from MR dating back from the early 1990s to the present. Treatment using a RSC brace involves a medical team collaboration among MR in Spain, Ortholutions in Germany, and the exclusive RSC brace treatment center. The RSC® brace is a scoliosis corset produced by computer technology according to the Ortholutions patented method. It is supplied exclusively to specialist firms trained for this. 

The concept is not only logical but also holistic, according to further development of the original Chêneau Brace by international scoliosis experts and Ortholutions. The model data bank grows continuously with the latest scientific knowledge. Each RSC® brace is produced according to the unique Ortholutions patented method. One of the many special features is the individual diagnosis and brace design for each individual patient. 

The main goal is to provide valuable treatment with a high rate of correction. All patients are seen and classified by two MDs, two CPOs and one physiotherapist. Every patient has their own database, where all data is stored and can be viewed and evaluated by the team or health insurance provider. 
